# Crystal structure of 1*H*,1′*H*-[2,2′-biimid­azol]-3-ium hydrogen tartrate hemi­hydrate

**DOI:** 10.1107/S160053681402371X

**Published:** 2014-10-31

**Authors:** Xiao-Li Gao, Li-Fang Bian, Shao-Wei Guo

**Affiliations:** a Department of Chemistry, Taiyuan Normal College, Taiyuan, Shanxi 030031, People’s Republic of China

**Keywords:** crystal structure, bi­imidazole, imidazolium, tartrate, hydrogen bonding

## Abstract

In the crystal of the title hydrated salt, C_6_H_7_N_4_^+^·C_4_H_5_O_6_^−^·0.5H_2_O, the bi­imidazole monocation, 1*H*,1′*H*-[2,2′-biimidazol]-3-ium, is hydrogen bonded, *via* N—H⋯O, O—H⋯O and O—H⋯N hydrogen bonds, to the hydrogen tartrate anion and the water mol­ecule, which is located on a twofold rotation axis, forming sheets parallel to (001). The sheets are linked *via* C—H⋯O hydrogen bonds, forming a three-dimensional structure. There are also C=O⋯π inter­actions present [O⋯π distances are 3.00 (9) and 3.21 (7) Å], involving the carbonyl O atoms and the imidazolium ring, which may help to consolidate the structure. In the cation, the dihedral angle between the rings is 11.6 (2)°.

## Related literature   

For background to the use of 2,2′-bi­imidazoles in crystal engineering, see: Shankar *et al.* (2013 ▶); Gulbransen & Fitchett (2012 ▶); Tadokoro & Nakasuji (2000 ▶). For similar structures, see: Liu & Zhu (2010 ▶); Gao *et al.* (2009 ▶); Li & Yang (2006 ▶); Mori & Miyoshi (2004 ▶).
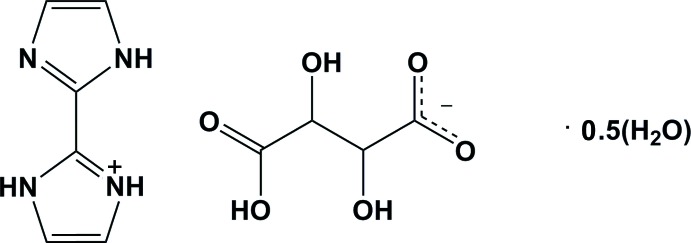


## Experimental   

### Crystal data  

C_4_H_5_O_6_^−^·C_6_H_7_N_4_^+^·0.5H_2_O
*M* *_r_* = 293.25
Monoclinic, 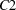

*a* = 19.3211 (13) Å
*b* = 4.8198 (2) Å
*c* = 16.1795 (10) Å
β = 122.694 (7)°
*V* = 1267.99 (13) Å^3^
*Z* = 4
Mo *K*α radiation
μ = 0.13 mm^−1^
*T* = 296 K
0.35 × 0.30 × 0.23 mm

### Data collection  

Bruker SMART diffractometer
Absorption correction: multi-scan (*SADABS*; Sheldrick, 2000 ▶) *T*_min_ = 0.956, *T*_max_ = 0.971
4238 measured reflections
2292 independent reflections
2100 reflections with *I* > 2σ(*I*)
*R*_int_ = 0.030

### Refinement  

*R*[*F* ^2^ > 2σ(*F* ^2^)] = 0.041
*wR*(*F* ^2^) = 0.099
*S* = 1.11
2292 reflections
202 parameters
1 restraint
H atoms treated by a mixture of independent and constrained refinement
Δρ_max_ = 0.16 e Å^−3^
Δρ_min_ = −0.27 e Å^−3^

### 

Data collection: *SMART* (Bruker, 2000 ▶); cell refinement: *SAINT* (Bruker, 2000 ▶); data reduction: *SAINT*; program(s) used to solve structure: *SHELXS97* (Sheldrick, 2008 ▶); program(s) used to refine structure: *SHELXL97* (Sheldrick, 2008 ▶); molecular graphics: *SHELXTL* (Sheldrick, 2008 ▶); software used to prepare material for publication: pubCIF (Westrip, 2010 ▶).

## Supplementary Material

Crystal structure: contains datablock(s) I, New_Global_Publ_Block. DOI: 10.1107/S160053681402371X/su5001sup1.cif

Structure factors: contains datablock(s) I. DOI: 10.1107/S160053681402371X/su5001Isup2.hkl

Supporting information file. DOI: 10.1107/S160053681402371X/su5001Isup3.cml

**Fig. 1.**
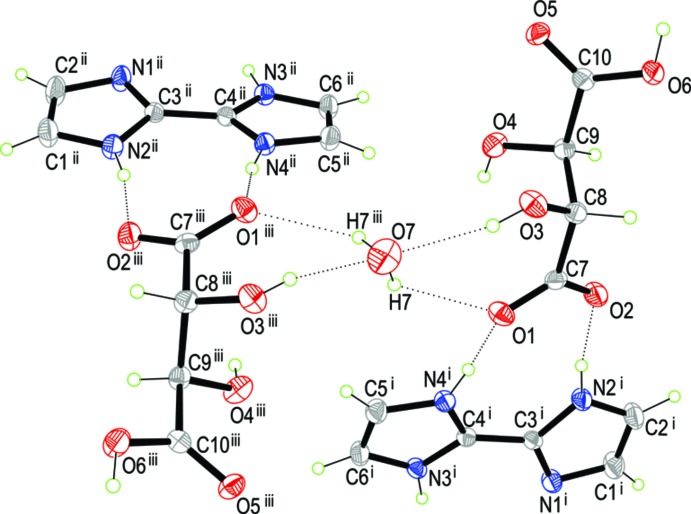
Molecular structure and atom labelling of the title compound, with displacement ellipsoids drawn at the 30% probability level. Dashed line indicates hydrogen bonds [see Table 1 for details; symmetry codes: (i) x, y + 1, z; (ii) x + 3/2, y + 3/2, z + 2; (iii) x + 3/2, y + 1/2, z + 2].

Click here for additional data file.x y z x y z x y z . DOI: 10.1107/S160053681402371X/su5001fig1.tif
Mol­ecular structure and atom labelling of the title compound, with displacement ellipsoids drawn at the 30% probability level. Dashed line indicates hydrogen bonds [see Table 1 for details; symmetry codes: (i) *x*, *y* + 1, *z*; (ii) *x* + 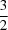, *y* + 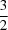, *z* + 2; (iii) *x* + 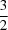, *y* + 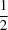, *z* + 2].

**Fig. 2.**
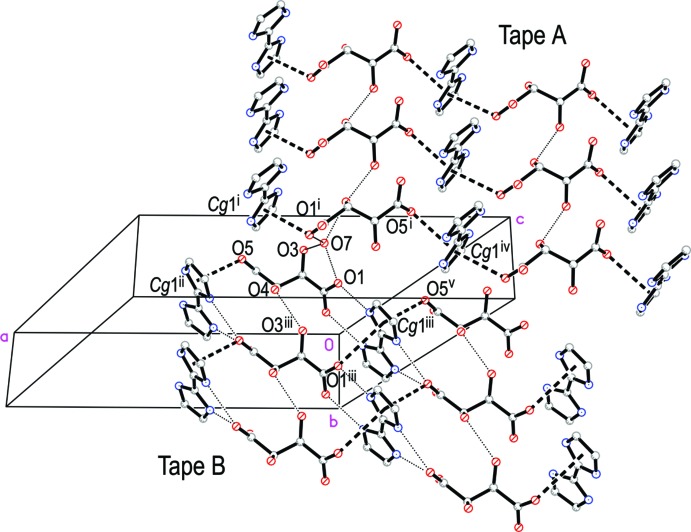
Partial crystal packing of the title compound, with the hydrogen bonds (dashed lines) and C═O···π interactions (dashed solid lines) between neighbouring tapes. Cg1 is the centroid of the C4/C5/C6/N3/N4 imidazole ring. [Symmetry codes: (i) x + 3/2, y + 1/2, z + 2; (ii) - x, y, - z; (iii) x, y + 1, z; (iv) x + 3/2, y - 3/2, z + 2; (v) - x - 1, y, - z.]

Click here for additional data file.x y z x y z x y z x y - z x y z . DOI: 10.1107/S160053681402371X/su5001fig2.tif
Partial crystal packing of the title compound, with the hydrogen bonds (dashed lines) and C=O⋯π inter­actions (dashed solid lines) between neighbouring tapes [symmetry codes: (i) *x* + 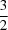, *y* + 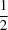, *z* + 2; (ii) − *x*, *y*, − *z*; (iii) *x*, *y* + 1, *z*; (iv) *x* + 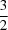, *y -* 3/2, *z* + 2; (v) − *x* − 1, *y*, − *z*].

CCDC reference: 1031345

Additional supporting information:  crystallographic information; 3D view; checkCIF report

## Figures and Tables

**Table 1:** Hydrogen-bond geometry (, )

*D*H*A*	*D*H	H*A*	*D* *A*	*D*H*A*
N2H2*A*O2^i^	0.92(3)	1.78(3)	2.683(3)	169(3)
N3H3*A*O5^ii^	0.94(3)	1.81(3)	2.729(3)	167(3)
N4H4*A*O1^i^	0.91(3)	1.73(3)	2.630(3)	167(3)
O3H3O7	0.83	2.05	2.871(2)	168
O4H4O3^iii^	0.91(4)	1.86(4)	2.761(3)	175(3)
O6H6*A*N1^iv^	0.82	1.79	2.598(3)	168
O7H7*A*O1	0.93	2.19	2.802(2)	123
C2H2O2^v^	0.93	2.35	3.215(3)	154
C5H5O4^vi^	0.93	2.55	3.415(3)	155
C6H6O5^vii^	0.93	2.37	3.205(3)	149

Symmetry codes: (i) 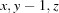; (ii) 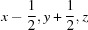; (iii) 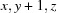; (iv) 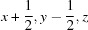; (v) 
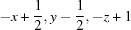
; (vi) 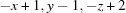; (vii) 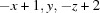.

## supplementary crystallographic information

### S1. Synthesis and crystallization

Di­imidazole (1.0 mmol) and tartaric acid (1.0 mmol) were dissolved in water (15 ml) by adding 1.2 ml of 2M HCl while stirring. The solution was left to stand at room temperature and colourless crystals of the title compound were obtained after several weeks.

### S1.1. Refinement

The NH H atoms and the water molecule H atom were located in a difference Fourier map and refined with U_iso_(H) = 1.2U_eq_(N,O). The O and C bound H atoms were placed in geometrically idealized positions with C—H = 0.93Å and O—H = 0.84 Å and constrained to refine with U_iso_(H) = 1.2U_eq_(O,C).

### S2. Comment

Supramolecular assemblies built by means of hydrogen bonding interactions have provided numerous materials with very attractive properties (Gulbransen & Fitchett, 2012; Shankar *et al.*, 2013). 2,2'-Biimidazole, H_2_biim, is not only a proton donor, but also a proton acceptor, so that it possesses five possible forms, viz. diprotonated (dication, H_4_biim^2+^), monoprotonated (monocation, H_3_biim^+^), dideprotonated (dianion, biim^2-^), monodeprotonated (monoanion, Hbiim^-^) (Tadokoro & Nakasuji, 2000; Mori & Miyoshi, 2004). Therefore, H_2_biim appears as an interesting molecular building block for the design of new multidimensional supramolecular arrangements, owing to its capacity to act as a donor or acceptor in the formation of hydrogen bonds (Li & Yang, 2006; Gao *et al.*, 2009; Liu & Zhu, 2010).

The fundamental asymmetric unit of compound (I), contains two monoprotonated biimidazolium cations, two tatrate anions and one water molecular, in which the two imidazole rings of biimidazole are little tortile with the dihedral number is 11.5°. Strong N—H···O and O—H···N hydrogen bonds link neighbour tatrate and biimidazolium moities, then O—H···O hydrogen bonds between water molecular and tatrates link them to form two different zigzag layers as shown in Fig. 1. Two groups of these parallel layers on a twofold rotation axis and invension centre forming a zigzag conformation, then further assemble to tapes *via* weak C=O···π (centroid of imidazolium ring) interaction arranged alternatively in three-dimensional structure as described in Table 1 and shown in Fig. 2.

### Crystal data

**Table:**

C_4_H_5_O_6_^−^·C_6_H_7_N_4_^+^·0.5H_2_O	*F*(000) = 612
*M**_r_* = 293.25	*D*_x_ = 1.536 Mg m^−^^3^
Monoclinic, *C*2	Mo *K*α radiation, λ = 0.71073 Å
Hall symbol: C 2y	Cell parameters from 2195 reflections
*a* = 19.3211 (13) Å	θ = 3.5–25.0°
*b* = 4.8198 (2) Å	µ = 0.13 mm^−^^1^
*c* = 16.1795 (10) Å	*T* = 296 K
β = 122.694 (7)°	Plate, colourless
*V* = 1267.99 (13) Å^3^	0.35 × 0.30 × 0.23 mm
*Z* = 4	

### Data collection

**Table:**

Bruker SMART diffractometer	2292 independent reflections
Radiation source: fine-focus sealed tube	2100 reflections with *I* > 2σ(*I*)
Graphite monochromator	*R*_int_ = 0.030
Detector resolution: 16.0733 pixels mm^-1^	θ_max_ = 25.5°, θ_min_ = 3.6°
phi and ω scans	*h* = −23→10
Absorption correction: multi-scan (*SADABS*; Sheldrick, 2000)	*k* = −5→5
*T*_min_ = 0.956, *T*_max_ = 0.971	*l* = −18→19
4238 measured reflections	

### Refinement

**Table:**

Refinement on *F*^2^	Primary atom site location: structure-invariant direct methods
Least-squares matrix: full	Secondary atom site location: difference Fourier map
*R*[*F*^2^ > 2σ(*F*^2^)] = 0.041	Hydrogen site location: inferred from neighbouring sites
*wR*(*F*^2^) = 0.099	H atoms treated by a mixture of independent and constrained refinement
*S* = 1.11	*w* = 1/[σ^2^(*F*_o_^2^) + (0.0364*P*)^2^ + 0.5619*P*] where *P* = (*F*_o_^2^ + 2*F*_c_^2^)/3
2292 reflections	(Δ/σ)_max_ < 0.001
202 parameters	Δρ_max_ = 0.16 e Å^−^^3^
1 restraint	Δρ_min_ = −0.27 e Å^−^^3^

### Special details

**Table:**

Geometry. All e.s.d.'s (except the e.s.d. in the dihedral angle between two l.s. planes) are estimated using the full covariance matrix. The cell e.s.d.'s are taken into account individually in the estimation of e.s.d.'s in distances, angles and torsion angles; correlations between e.s.d.'s in cell parameters are only used when they are defined by crystal symmetry. An approximate (isotropic) treatment of cell e.s.d.'s is used for estimating e.s.d.'s involving l.s. planes.
Refinement. Refinement of *F*^2^ against ALL reflections. The weighted *R*-factor *wR* and goodness of fit *S* are based on *F*^2^, conventional *R*-factors *R* are based on *F*, with *F* set to zero for negative *F*^2^. The threshold expression of *F*^2^ > σ(*F*^2^) is used only for calculating *R*-factors(gt) *etc*. and is not relevant to the choice of reflections for refinement. *R*-factors based on *F*^2^ are statistically about twice as large as those based on *F*, and *R*- factors based on ALL data will be even larger.The line _refine_ls_abs_structure_Flack -1.5 (15) has been removed. According to the comment in an absolute configuration has been assigned, obtained using *x* by least-squares refinement. There is too high standard uncertainty on *x* and no information available that the assigned value is confirmed by the diffraction measurements.

### Fractional atomic coordinates and isotropic or equivalent isotropic displacement parameters (Å^2^)

**Table:**

	*x*	*y*	*z*	*U*_iso_*/*U*_eq_	
C1	0.12363 (19)	0.3256 (7)	0.54798 (19)	0.0501 (8)	
H1	0.0811	0.4221	0.4950	0.060*	
C2	0.17427 (17)	0.1439 (8)	0.54276 (19)	0.0465 (8)	
H2	0.1729	0.0925	0.4865	0.056*	
C3	0.20830 (14)	0.1770 (6)	0.69462 (16)	0.0287 (6)	
C4	0.25343 (14)	0.1445 (5)	0.80032 (16)	0.0266 (5)	
C5	0.33858 (17)	−0.0022 (6)	0.95007 (19)	0.0385 (7)	
H5	0.3788	−0.1057	1.0029	0.046*	
C6	0.29783 (17)	0.2162 (6)	0.95549 (18)	0.0380 (7)	
H6	0.3044	0.2923	1.0121	0.046*	
N1	0.14434 (14)	0.3465 (5)	0.64335 (15)	0.0400 (6)	
N2	0.22801 (13)	0.0497 (5)	0.63601 (15)	0.0357 (5)	
N3	0.24455 (13)	0.3045 (5)	0.86056 (14)	0.0307 (5)	
N4	0.31045 (13)	−0.0455 (5)	0.85300 (15)	0.0307 (5)	
H2A	0.2689 (17)	−0.079 (6)	0.6544 (19)	0.037*	
H3A	0.2032 (17)	0.440 (6)	0.8388 (18)	0.037*	
H4A	0.3337 (16)	−0.172 (7)	0.8333 (19)	0.037*	
C7	0.38284 (14)	0.5315 (5)	0.74083 (18)	0.0295 (6)	
C8	0.44290 (15)	0.3252 (6)	0.74053 (18)	0.0308 (6)	
C9	0.51381 (14)	0.4868 (5)	0.74572 (17)	0.0288 (6)	
H9	0.4916	0.6076	0.6881	0.035*	
C10	0.57380 (15)	0.2829 (6)	0.74598 (17)	0.0299 (6)	
O1	0.37823 (11)	0.5416 (4)	0.81509 (13)	0.0379 (5)	
O2	0.34295 (11)	0.6789 (4)	0.66602 (12)	0.0436 (5)	
O3	0.47460 (11)	0.1317 (4)	0.81901 (13)	0.0382 (5)	
H3	0.4744	0.1836	0.8679	0.046*	
O4	0.55700 (11)	0.6495 (4)	0.83171 (14)	0.0402 (5)	
O5	0.64334 (10)	0.2498 (4)	0.81780 (12)	0.0353 (5)	
O6	0.54328 (11)	0.1477 (5)	0.66429 (13)	0.0475 (5)	
H6A	0.5803	0.0673	0.6635	0.057*	
H4	0.5313 (19)	0.807 (8)	0.832 (2)	0.057*	
H8	0.4108 (19)	0.231 (7)	0.676 (2)	0.057*	
O7	0.5000	0.3371 (9)	1.0000	0.0714 (10)	
H7A	0.4667	0.4934	0.9729	0.086*	

### Atomic displacement parameters (Å^2^)

**Table:**

	*U*^11^	*U*^22^	*U*^33^	*U*^12^	*U*^13^	*U*^23^
C1	0.0480 (17)	0.063 (2)	0.0286 (13)	0.0215 (17)	0.0139 (13)	0.0062 (15)
C2	0.0506 (17)	0.0589 (19)	0.0278 (13)	0.0134 (17)	0.0196 (12)	0.0000 (14)
C3	0.0276 (12)	0.0321 (13)	0.0280 (12)	0.0028 (12)	0.0161 (10)	0.0002 (12)
C4	0.0262 (12)	0.0257 (12)	0.0282 (11)	0.0020 (11)	0.0149 (10)	0.0017 (11)
C5	0.0380 (14)	0.0426 (16)	0.0297 (13)	0.0072 (13)	0.0149 (11)	0.0046 (12)
C6	0.0405 (15)	0.0437 (17)	0.0273 (12)	0.0023 (13)	0.0168 (12)	−0.0015 (12)
N1	0.0373 (12)	0.0501 (14)	0.0286 (11)	0.0179 (12)	0.0152 (10)	0.0038 (11)
N2	0.0348 (12)	0.0400 (13)	0.0305 (11)	0.0110 (11)	0.0165 (10)	−0.0011 (10)
N3	0.0327 (11)	0.0322 (12)	0.0293 (11)	0.0050 (10)	0.0180 (9)	0.0005 (9)
N4	0.0318 (11)	0.0310 (12)	0.0295 (11)	0.0067 (10)	0.0167 (9)	0.0033 (9)
C7	0.0245 (12)	0.0283 (13)	0.0354 (13)	−0.0009 (11)	0.0160 (10)	−0.0059 (11)
C8	0.0300 (13)	0.0297 (14)	0.0312 (12)	0.0041 (11)	0.0155 (11)	−0.0018 (11)
C9	0.0299 (12)	0.0254 (13)	0.0286 (12)	0.0069 (11)	0.0141 (10)	0.0028 (11)
C10	0.0322 (13)	0.0296 (13)	0.0318 (13)	−0.0007 (11)	0.0198 (12)	−0.0003 (11)
O1	0.0442 (11)	0.0357 (10)	0.0423 (10)	0.0090 (9)	0.0290 (9)	0.0043 (8)
O2	0.0412 (11)	0.0545 (13)	0.0332 (9)	0.0233 (11)	0.0190 (8)	0.0074 (10)
O3	0.0501 (11)	0.0284 (10)	0.0445 (10)	0.0103 (9)	0.0311 (9)	0.0066 (9)
O4	0.0355 (10)	0.0283 (9)	0.0462 (10)	0.0036 (9)	0.0151 (8)	−0.0117 (9)
O5	0.0268 (9)	0.0365 (10)	0.0347 (9)	0.0052 (8)	0.0114 (8)	−0.0045 (8)
O6	0.0349 (10)	0.0641 (14)	0.0352 (9)	0.0139 (10)	0.0135 (8)	−0.0140 (10)
O7	0.064 (2)	0.078 (3)	0.061 (2)	0.000	0.0266 (18)	0.000

### Geometric parameters (Å, º)

**Table:**

C1—C2	1.350 (4)	N4—H4A	0.91 (3)
C1—N1	1.373 (3)	C7—O2	1.247 (3)
C1—H1	0.9300	C7—O1	1.253 (3)
C2—N2	1.367 (3)	C7—C8	1.530 (4)
C2—H2	0.9300	C8—O3	1.421 (3)
C3—N1	1.333 (3)	C8—C9	1.538 (4)
C3—N2	1.346 (3)	C8—H8	0.99 (3)
C3—C4	1.449 (3)	C9—O4	1.412 (3)
C4—N3	1.325 (3)	C9—C10	1.518 (3)
C4—N4	1.328 (3)	C9—H9	0.9800
C5—C6	1.346 (4)	C10—O5	1.222 (3)
C5—N4	1.372 (3)	C10—O6	1.295 (3)
C5—H5	0.9300	O3—H3	0.8309
C6—N3	1.375 (3)	O4—H4	0.91 (4)
C6—H6	0.9300	O6—H6A	0.8200
N2—H2A	0.92 (3)	O7—H7A	0.9324
N3—H3A	0.94 (3)		
			
C2—C1—N1	109.7 (2)	C4—N4—C5	107.9 (2)
C2—C1—H1	125.2	C4—N4—H4A	129.2 (17)
N1—C1—H1	125.2	C5—N4—H4A	122.4 (17)
C1—C2—N2	106.9 (2)	O2—C7—O1	125.6 (2)
C1—C2—H2	126.6	O2—C7—C8	116.0 (2)
N2—C2—H2	126.6	O1—C7—C8	118.4 (2)
N1—C3—N2	111.2 (2)	O3—C8—C7	112.7 (2)
N1—C3—C4	124.5 (2)	O3—C8—C9	110.02 (19)
N2—C3—C4	124.3 (2)	C7—C8—C9	109.0 (2)
N3—C4—N4	108.8 (2)	O3—C8—H8	111 (2)
N3—C4—C3	124.8 (2)	C7—C8—H8	105.0 (19)
N4—C4—C3	126.4 (2)	C9—C8—H8	108.7 (18)
C6—C5—N4	108.0 (2)	O4—C9—C10	108.24 (19)
C6—C5—H5	126.0	O4—C9—C8	111.7 (2)
N4—C5—H5	126.0	C10—C9—C8	109.19 (19)
C5—C6—N3	106.3 (2)	O4—C9—H9	109.2
C5—C6—H6	126.8	C10—C9—H9	109.2
N3—C6—H6	126.8	C8—C9—H9	109.2
C3—N1—C1	105.3 (2)	O5—C10—O6	124.4 (2)
C3—N2—C2	106.9 (2)	O5—C10—C9	122.2 (2)
C3—N2—H2A	126.9 (17)	O6—C10—C9	113.4 (2)
C2—N2—H2A	126.1 (17)	C8—O3—H3	115.5
C4—N3—C6	109.0 (2)	C9—O4—H4	116 (2)
C4—N3—H3A	123.3 (16)	C10—O6—H6A	109.5
C6—N3—H3A	127.3 (16)		
			
N1—C1—C2—N2	−0.3 (4)	N3—C4—N4—C5	−0.2 (3)
N1—C3—C4—N3	−9.7 (4)	C3—C4—N4—C5	179.2 (3)
N2—C3—C4—N3	166.9 (3)	C6—C5—N4—C4	0.1 (3)
N1—C3—C4—N4	170.9 (3)	O2—C7—C8—O3	−169.2 (2)
N2—C3—C4—N4	−12.4 (4)	O1—C7—C8—O3	11.6 (3)
N4—C5—C6—N3	0.1 (3)	O2—C7—C8—C9	68.4 (3)
N2—C3—N1—C1	−0.6 (3)	O1—C7—C8—C9	−110.8 (3)
C4—C3—N1—C1	176.5 (3)	O3—C8—C9—O4	−64.0 (2)
C2—C1—N1—C3	0.5 (4)	C7—C8—C9—O4	60.0 (2)
N1—C3—N2—C2	0.4 (3)	O3—C8—C9—C10	55.7 (3)
C4—C3—N2—C2	−176.7 (3)	C7—C8—C9—C10	179.7 (2)
C1—C2—N2—C3	0.0 (4)	O4—C9—C10—O5	9.8 (3)
N4—C4—N3—C6	0.3 (3)	C8—C9—C10—O5	−112.0 (3)
C3—C4—N3—C6	−179.1 (3)	O4—C9—C10—O6	−171.4 (2)
C5—C6—N3—C4	−0.3 (3)	C8—C9—C10—O6	66.8 (3)

### Hydrogen-bond geometry (Å, º)

**Table:**

*D*—H···*A*	*D*—H	H···*A*	*D*···*A*	*D*—H···*A*
N2—H2*A*···O2^i^	0.92 (3)	1.78 (3)	2.683 (3)	169 (3)
N3—H3*A*···O5^ii^	0.94 (3)	1.81 (3)	2.729 (3)	167 (3)
N4—H4*A*···O1^i^	0.91 (3)	1.73 (3)	2.630 (3)	167 (3)
O3—H3···O7	0.83	2.05	2.871 (2)	168
O4—H4···O3^iii^	0.91 (4)	1.86 (4)	2.761 (3)	175 (3)
O6—H6*A*···N1^iv^	0.82	1.79	2.598 (3)	168
O7—H7*A*···O1	0.93	2.19	2.802 (2)	123
C2—H2···O2^v^	0.93	2.35	3.215 (3)	154
C5—H5···O4^vi^	0.93	2.55	3.415 (3)	155
C6—H6···O5^vii^	0.93	2.37	3.205 (3)	149

Symmetry codes: (i) *x*, *y*−1, *z*; (ii) *x*−1/2, *y*+1/2, *z*; (iii) *x*, *y*+1, *z*; (iv) *x*+1/2, *y*−1/2, *z*; (v) −*x*+1/2, *y*−1/2, −*z*+1; (vi) −*x*+1, *y*−1, −*z*+2; (vii) −*x*+1, *y*, −*z*+2.
