# Supplementary figures and images for: Crystal structure of 1H,1′H-[2,2′-biimid­azol]-3-ium hydrogen tartrate hemi­hydrate
Source: Acta Crystallogr Sect E Struct Rep Online. 2014 Oct 31;70(Pt 11):o1221–2. doi: 10.1107/S160053681402371X (PMC4257278; doi:10.1107/S160053681402371X)

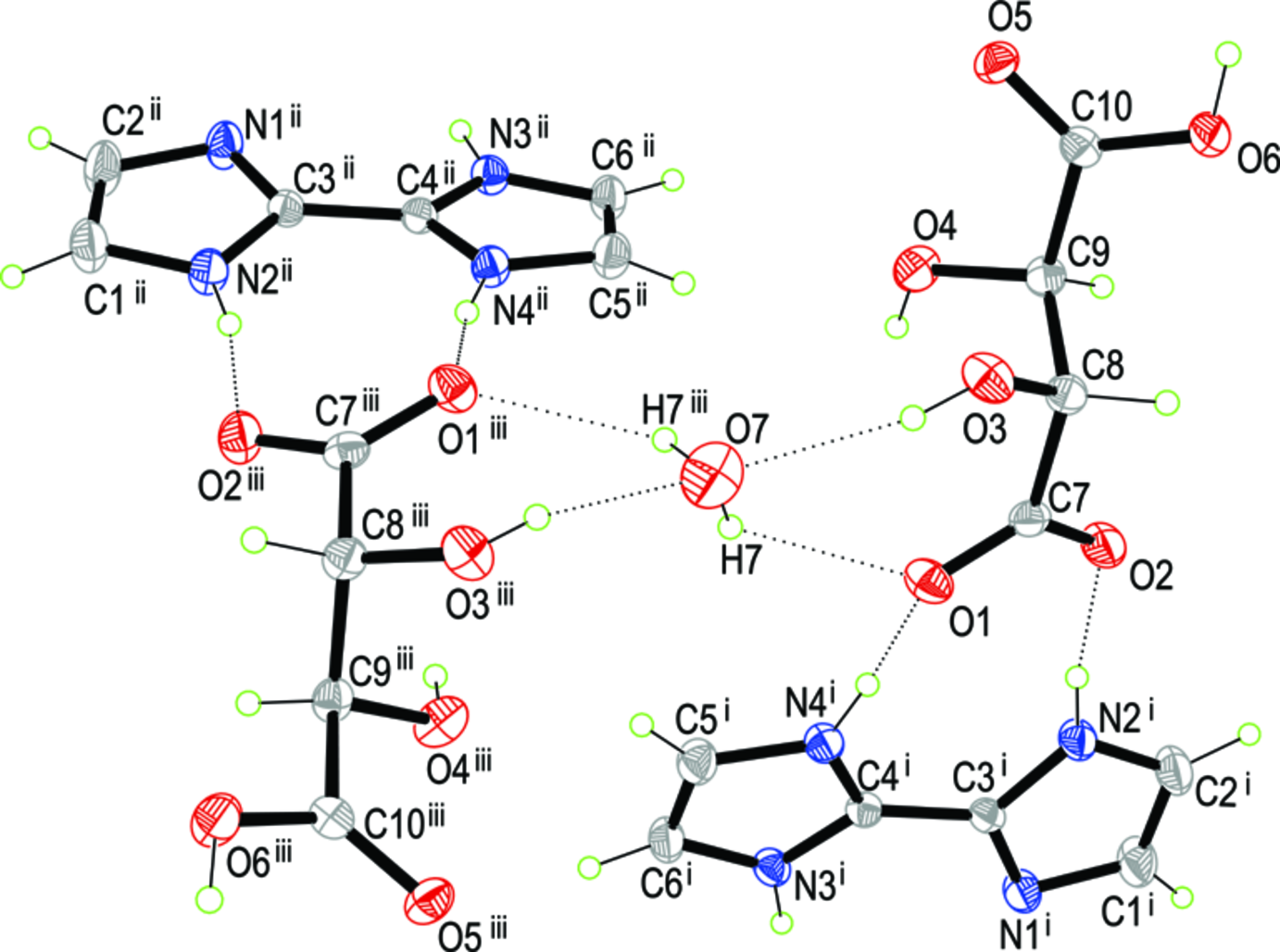

Supplement: Supplementary file 4 [file e-70-o1221-fig1.tif]

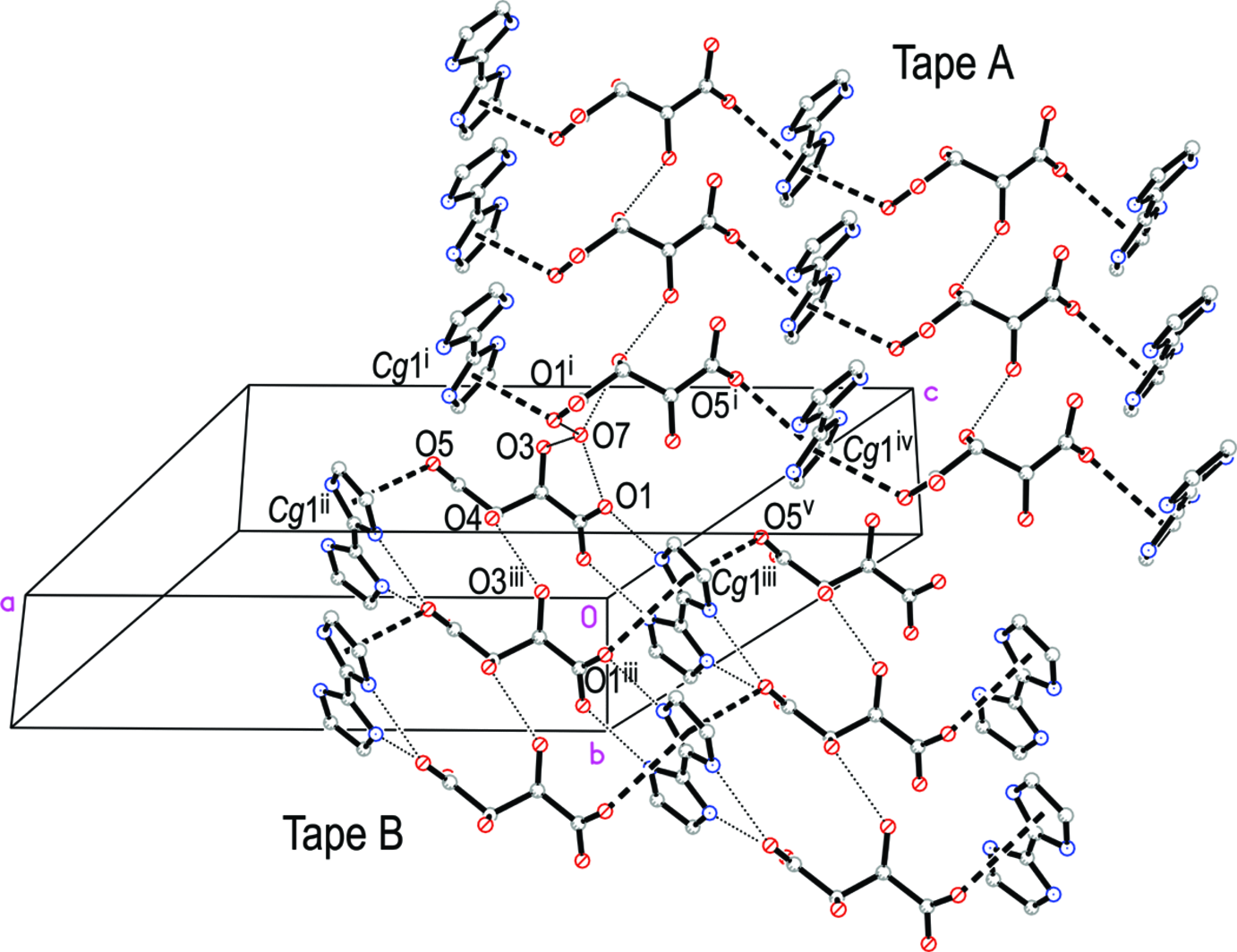

Supplement: Supplementary file 5 [file e-70-o1221-fig2.tif]
